# Nevus Sebaceus Arising Within a Scalp Whorl of a Healthy Male Neonate

**DOI:** 10.7759/cureus.30094

**Published:** 2022-10-09

**Authors:** Carli P Whittington, Simran Kalsi, Keith W Morley, Melanie R Bui

**Affiliations:** 1 Department of Medicine, Division of Dermatology, University of Vermont Medical Center, Burlington, USA; 2 Department of Medicine, Division of Dermatology, Larner College of Medicine, University of Vermont, Burlington, USA

**Keywords:** neonate, benign, organoid nevus, hair whorl, scalp whorl, nevus sebaceus

## Abstract

Nevus sebaceus (NS) and scalp whorl are both benign congenital findings that have not previously been reported to occur simultaneously. In most cases, the isolated finding of a single, classic-appearing NS or a single hair whorl can be followed clinically with observation. However, the number of lesions, distribution, and size of NS along with atypical placement of a scalp hair whorl can indicate an underlying syndrome or even underlying cranial abnormalities. We present a unique case of NS arising within a hair whorl on the vertex scalp of an otherwise healthy male neonate. After ultrasound showed no vascular malformations or proliferations and no cranial extension at the site, the lesion was later treated with surgical excision at six months old per the parents’ preference, thus allowing for histologic confirmation of NS. Additionally, we discuss herein the diagnostic implications, recommendations for work-up, and treatment options of NS.

## Introduction

Nevus sebaceus (NS), also known as nevus sebaceus of Jadassohn or organoid nevus, is a benign adnexal malformation that commonly presents at birth involving the head, scalp, or face. NS is susceptible to hormone-mediated changes during puberty and can develop secondary neoplasms in adulthood. Management for NS has evolved over the years, now typically favoring clinical observation in infancy and early childhood, followed by complete excision during adolescence before puberty [1-7]. If multiple lesions are present and/or a single lesion is very large in size, NS may be associated with certain syndromes, such as Schimmelpenning syndrome or phakomatosis pigmentokeratotica, both of which can present with extracutaneous defects [5]. Hair whorls are typically located on the vertex scalp and present as circular arrangements of hair. Atypically placed, or absent, scalp whorls have been associated with abnormal brain development [8-14]. In these cases, further imaging and work-up may be necessitated to rule out intracranial abnormalities. Herein we report an NS arising on the vertex scalp of a healthy male neonate within a scalp whorl.

## Case presentation

**Figure 1:**
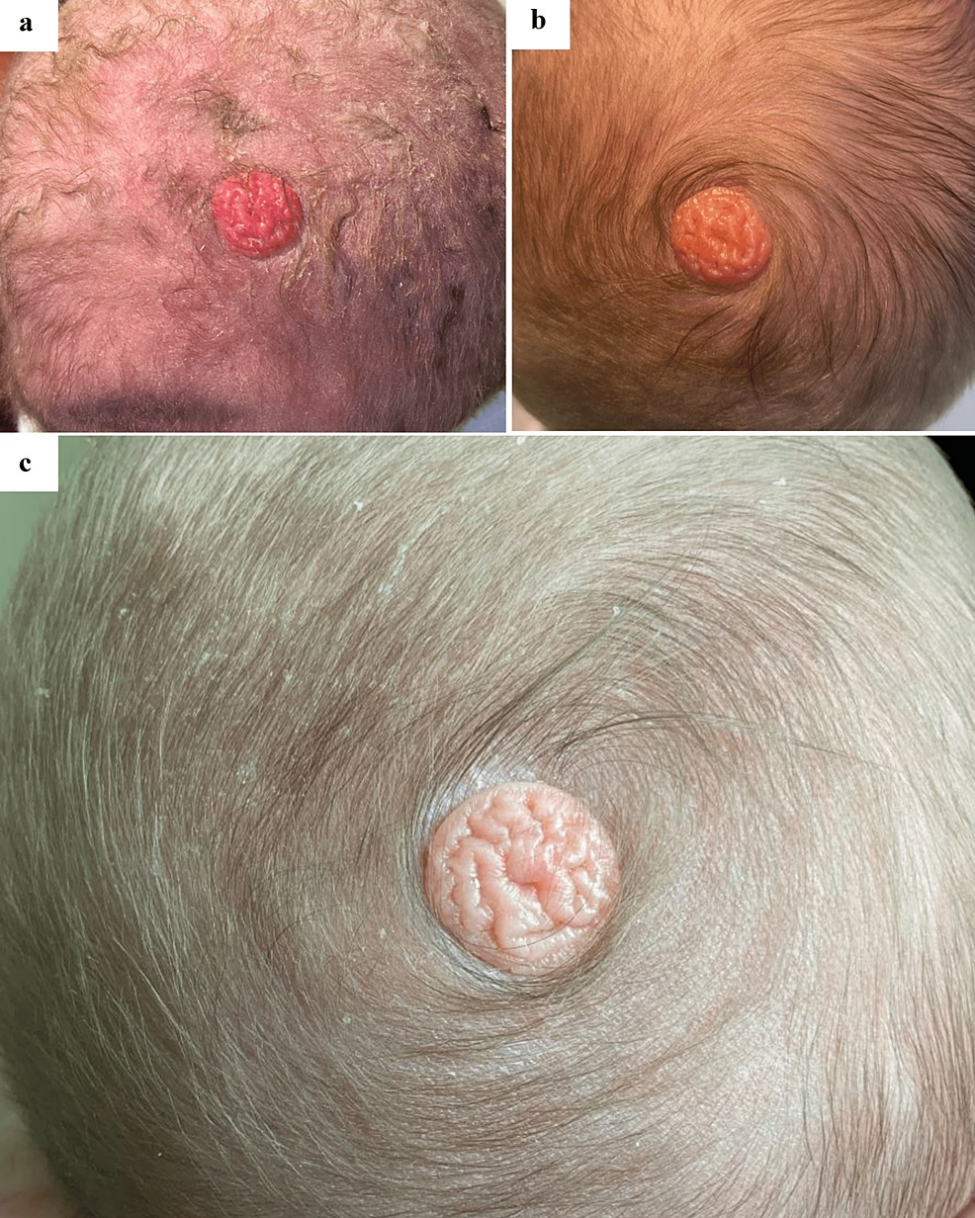
(a) red cerebriform plaque on the vertex scalp at birth, (b) pink cerebriform plaque on the vertex scalp arising within a hair whorl at day one of life, (c) yellow-pink cerebriform plaque on the vertex scalp arising within a hair whorl at month one of life.

A one-day-old healthy male neonate presented with a solitary red, cerebriform plaque on the vertex scalp arising within a single hair whorl that was noted at birth (Figure 1a) and day one of life (Figure 1b). Physical examination revealed no other cutaneous birthmarks present on the face or body. Due to diagnostic uncertainty, an ultrasound was obtained which showed a soft tissue mass situated between the dermis and subdermal fat with minimal internal vascular flow. There was no invasion into the galea aponeuroses and no connection to or disruption of the periosteum. At one-month-old, the patient returned to dermatology clinic and the cerebriform lesion was observed to be more yellowish-orange in color (Figure 1c). Per his parents’ preference, the patient underwent surgical excision with a pediatric plastic surgeon at six months of age and tolerated the procedure without issues. 

**Figure 2:**
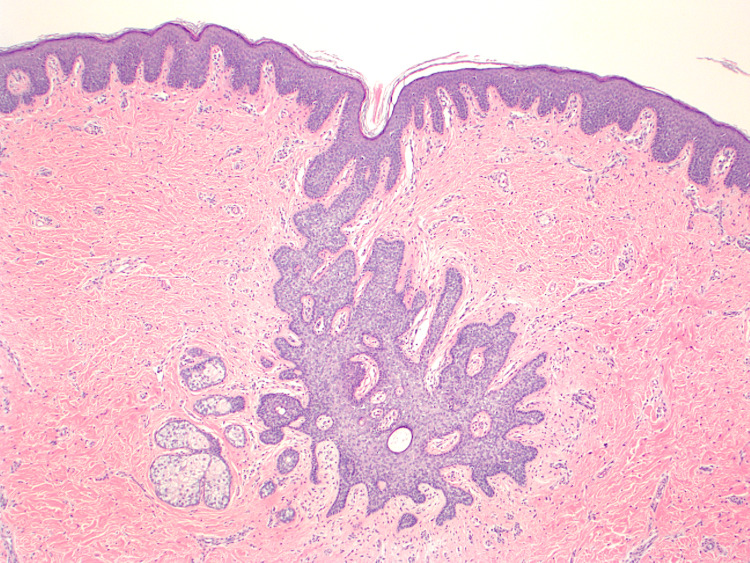
Diminutive pilosebaceous unit with abortive hair follicles and smaller than normal sebaceous glands (H&E, 4x magnification). H&E = hematoxylin and eosin

The surgical specimen was submitted for histologic analysis, which showed numerous immature, malformed sebaceous units in the superficial dermis, tiny misshapen hair follicles, and an acanthotic and papillomatous epidermis (Figure 2). The surrounding scalp skin showed normal folliculosebaceous units with terminal hairs arising from the subcutaneous fat. Given these findings, the patient was diagnosed with NS.

## Discussion

NS is a congenital, benign adnexal hamartoma seen in about 0.3% of all neonates. Though it can develop at almost any body site, the most common sites are the head, scalp, and face. Much less commonly, NS can be extensive and cover multiple body regions, raising concern for an associated syndrome [1]. NS is typically asymptomatic, present at birth, and appears as a subtle light yellow to skin-colored patch or thin plaque on the scalp or face. If linear in morphology, NS may be distributed along the lines of Blaschko. Rarely, NS can present as a cerebriform, exophytic plaque or tumor [2-5].

During puberty, NS undergoes hormone-mediated changes and becomes more raised (thicker) and verrucous (wart-like) in texture, often raising cosmetic concerns for patients and/or their parents. If present on hair-bearing regions, NS can cause focal alopecia with normal hair growing around it. If a single NS is large in size, covers a large body surface area, and/or there are multiple individual NS lesions, they may be associated with certain underlying syndromes including Schimmelpenning syndrome and phakomatosis pigmentokeratotica. Secondary neoplasms arising within NS have been reported, including trichoblastoma, syringocystadenoma papilliferum, and even more rarely malignant neoplasms such as basal cell carcinoma, squamous cell carcinoma, and sebaceous carcinoma. Studies have shown that different mutations in the HRAS, KRAS, and FGFR2 genes can be associated with NS [5]. Diagnosis of NS can be made clinically or confirmed with skin biopsy, especially if the lesion does not have a classic appearance on physical examination.

On histology, NS displays follicular, sebaceous, and sometimes apocrine components; however, the exact features depend on whether the patient has gone through puberty or not. In pre-pubertal NS lesions, there are numerous, albeit diminutive, malformed folliculosebaceous units within the superficial dermis with abortive hair follicles and small sebaceous glands. In post-puberty NS lesions, the epidermis is more acanthotic, papillomatous, and verrucous with fibroplasia in the papillary dermis [7]. Furthermore, post-puberty NS lesions have enlarged sebaceous lobules, dilated apocrine glands, and underlying lesional follicular units that are small and distorted.

In terms of the natural course for NS, the lesion does not usually involute spontaneously. Management for NS has evolved over the years, now typically favoring clinical observation in infancy and early childhood, followed by complete excision during adolescence when the procedure can be performed with local anesthesia alone. The clinical differential diagnosis for NS includes keratinocyte epidermal nevus, inflammatory linear verrucous epidermal nevus, nevus comedonicus, nevus lipomatosis, Becker’s nevus, connective tissue nevus (such as collagenoma and elastoma), and encephalocele.

A hair whorl is a circular pattern of hair growth on the scalp that can spiral clockwise or counterclockwise [8]. Most commonly, a hair whorl involves the parietal or vertex scalp and can be located in the midline, or to the left or right of the midline. A single hair whorl or multiple hair whorls may be present. While hair whorls can be a normal feature of the human scalp and are mostly seen in healthy children without underlying issues, some case reports have described atypically placed, or even absent, scalp whorls being associated with underlying cranial or neurologic anomalies such as abnormally formed skull or incomplete brain development, or even associated with syndromes like neurofibromatosis type 1 [1, 9-14]. Similarly, a “hair collar sign” can also indicate the presence of an underlying cranial defect but is defined as a ring of hypertrichosis around the periphery of a lesion rather than a spiraling growth pattern of hair [15]. The hair collar sign is usually associated with localized congenital aplasia cutis on the scalp. Taken together, the findings of NS arising within a hair whorl should warrant consideration for further investigation to assess for any potential intracranial extensions. This should be performed in conjunction with a total body skin examination assessing for quantity and size of NS lesions. As NS are due to post-zygotic mutations, family history is unfortunately not revealing in the vast majority of cases.

## Conclusions

While there have only been a few reports of NS presenting as a cerebriform plaque, there have been no known cases to date in the literature describing a cerebriform-like NS specifically arising within a hair whorl on the scalp of a healthy neonate. Given the reported associations with underlying skull or brain abnormalities, the performance of imaging such as ultrasound should be considered prior to any surgical procedure to rule out connection with the central nervous system or bony attachment. As in this patient's case, histology can confirm the diagnosis of NS, especially if the diagnosis is in question. Additionally, a thorough skin examination should be performed to rule out the presence of large NS lesions and/or multiple individual NS lesions, as these are known to be associated with certain syndromes, including Schimmelpenning syndrome and phakomatosis pigmentokeratotica.
